# The Immune Profile of Pituitary Adenomas and a Novel Immune Classification for Predicting Immunotherapy Responsiveness

**DOI:** 10.1210/clinem/dgaa449

**Published:** 2020-07-12

**Authors:** Zihao Wang, Xiaopeng Guo, Lu Gao, Kan Deng, Wei Lian, Xinjie Bao, Ming Feng, Lian Duan, Huijuan Zhu, Bing Xing

**Affiliations:** 1 Department of Neurosurgery, Peking Union Medical College Hospital, Chinese Academy of Medical Sciences and Peking Union Medical College, Beijing, P. R. China; 2 Key Laboratory of Endocrinology of Ministry of Health, Peking Union Medical College Hospital, Chinese Academy of Medical Sciences and Peking Union Medical College, Beijing, P. R. China; 3 China Pituitary Disease Registry Center, Beijing, P. R. China; 4 China Pituitary Adenoma Specialist Council, Beijing, P. R. China; 5 Department of Endocrinology, Peking Union Medical College Hospital, Chinese Academy of Medical Sciences and Peking Union Medical College, Beijing, P. R. China

**Keywords:** Pituitary tumors, tumor-infiltrating immune cells, immune checkpoint molecules, immune classification, immunotherapy responsiveness

## Abstract

**Context:**

The tumor immune microenvironment is associated with clinical outcomes and immunotherapy responsiveness.

**Objective:**

To investigate the intratumoral immune profile of pituitary adenomas (PAs) and its clinical relevance and to explore a novel immune classification for predicting immunotherapy responsiveness.

**Design, Patients, and Methods:**

The transcriptomic data from 259 PAs and 20 normal pituitaries were included for analysis. The ImmuCellAI algorithm was used to estimate the abundance of 24 types of tumor-infiltrating immune cells (TIICs) and the expression of immune checkpoint molecules (ICMs).

**Results:**

The distributions of TIICs differed between PAs and normal pituitaries and varied among PA subtypes. T cells dominated the immune microenvironment across all subtypes of PAs. The tumor size and patient age were correlated with the TIIC abundance, and the ubiquitin-specific protease 8 (USP8) mutation in corticotroph adenomas influenced the intratumoral TIIC distributions. Three immune clusters were identified across PAs based on the TIIC distributions. Each cluster of PAs showed unique features of ICM expression that were correlated with distinct pathways related to tumor development and progression. CTLA4/CD86 expression was upregulated in cluster 1, whereas programmed cell death protein 1/programmed cell death 1 ligand 2 (PD1/PD-L2) expression was upregulated in cluster 2. Clusters 1 and 2 exhibited a “hot” immune microenvironment and were predicted to exhibit higher immunotherapy responsiveness than cluster 3, which exhibited an overall “cold” immune microenvironment.

**Conclusions:**

We summarized the immune profile of PAs and identified 3 novel immune clusters. These findings establish a foundation for further immune studies on PAs and provide new insights into immunotherapy strategies for PAs.

Pituitary adenomas (PAs) are the second most common primary tumors of the central nervous system, with an annual incidence of 4 per 100 000 and a prevalence of 37 to 116 per 100 000 (1, 2). These tumors are classified clinically as secretory adenomas or nonfunctioning adenomas and classified radiologically as microadenomas (<10 mm) or macroadenomas (≥10 mm). The tumor mass effect, secondary hypopituitarism, and complications due to hormonal hypersecretion significantly reduce the quality of life and increase the mortality of patients with PAs (3-5). Most PAs are noninvasive and slow growing and are primarily treated by transsphenoidal surgery or medical treatment (eg, cabergoline and dopamine agonists). However, the gross total resection rate of PAs is only 66% to 78% (6, 7), with an overall remission rate of 40% to 70% for macroadenomas and 80% to 90% for microadenomas (8). Repeated surgery and radiotherapy are treatment options for recurrent PAs (9, 10). However, approximately 2.5% to 10% of PAs exhibit aggressive behavior, show repeated recurrence, and resist conventional treatments, and even temozolomide and these refractory PAs significantly increase the morbidity and mortality (9, 11). Based on the more in-depth understanding of tumor immunity that has been obtained in recent years, immunotherapy might serve as a promising alternative therapy for refractory tumors (12, 13).

The mechanisms underlying the development, progression, and chemoresistance of PAs have not been clearly elucidated. An unfavorable PA phenotype is defined not only by the intrinsic activity of tumor cells but also by the infiltrated immune cells in the tumor microenvironment (TME) (13, 14). Cytotoxic T-lymphocyte-associated antigen 4 (CTLA4) and programmed cell death protein 1 (PD1) are 2 main targeted immune-inhibitory checkpoints of T cells in the TME, and anti-CTLA4 therapy was the first approved immunotherapy to demonstrate benefits on the survival of patients with metastatic melanoma (15). An improved understanding of the immune characteristics of the TME and novel classifications based on different immune features might provide insights into the mechanisms of different immunotherapy responses and serve as resources for future targeted studies (16, 17). Several studies have investigated intratumoral T-cell infiltration and programmed death-ligand 1 (PD-L1) expression in PAs (13, 18, 19). Iacovazzo et al (19) demonstrated that a lower number of CD8^+^ T cells is related to both the invasion of PAs to the cavernous sinus and resistance to treatment. Studies conducted by Mei et al (18) and Wang et al (13) concluded that PD-L1 expression is higher in functioning PAs and correlated with tumor aggressiveness. Yeung et al (20) recently found that most PAs comprise macrophages and T cells and that each PA subtype has a unique immune infiltration pattern. However, although the above-mentioned studies provide an initial view of the distributions of tumor-infiltrating immune cells (TIICs) in PAs, our understanding of the immune profile of PAs remains very limited, and the clinical relevance of immune patterns remains largely unexplored.

Transcriptomic data have been used to study the TME and to estimate the distribution of TIICs in various tumors (21-25). Immune cell abundance identifier (ImmuCellAI) is a recently developed novel algorithm that uses gene set signatures to estimate the abundances of 24 types of immune cells from transcriptomic data, including ribonucleic acid (RNA) sequencing and microarray data (26). Compared with other known algorithms developed for enumerating immune cells from transcriptomic data (27-30), ImmuCellAI has the unique advantage of focusing on the specific T-cell subsets that play essential roles in tumor initiation and progression (26, 31). We hypothesize that this centralized analysis of T cells and related immune molecules could provide specific information for predicting responses to the current anti-PD1 and anti-CTLA4 therapies.

This study aimed to investigate the overall immune landscape of PAs and its clinical relevance and to identify novel immune classifications in the TME of PAs. First, we collected and analyzed the transcriptomic data from 140 PAs and 20 normal pituitaries using the ImmuCellAI algorithm and described both the distributions of 24 types of TIICs in PAs and details on the immune features of the 5 histological subtypes. Then we explored the effects of clinical and pathological parameters on the TME of PAs, including the effect of the ubiquitin-specific protease 8 (USP8) mutation on the TIICs of corticotroph PAs and the effect of treatment with somatostatin analog (SSA) on the TIICs of somatotroph PAs. In addition, we characterized 3 separate immune clusters in the TME of PAs that were independent of the histological subtype and presented different distribution patterns of immune cells and checkpoints; these clusters might help predict the response of PAs to targeted immunotherapies. We subsequently enrolled 119 more patients with PAs as a validation cohort for verifying the above results.

## Materials and Methods

### Data retrieval and processing

After extensive data screening, the transcriptomic profiles of 140 PA samples and 20 normal pituitary samples were downloaded from the Gene Expression Omnibus (GEO) database, and 134 PA samples were downloaded from the ArrayExpress database. The data were normalized using the “normalizeBetweenArray” function with the “SVA” and “LIMMA” packages in R software to remove batch effects and other unwanted variations (32). The details of the enrolled datasets are summarized in Supplementary Table 1 (33). Among the downloaded datasets, GSE136781 (34), GSE132982 (35), and E-MTAB-7768 (36) were profiled by RNA sequencing, whereas GSE119063, GSE93825 (37), GSE51618, GSE46311 (38), and GSE26966 (39) were profiled using microarrays. The 140 PA samples from the GEO database were divided into 5 subtypes as follows: 52 (37.1%) corticotroph adenomas, 16 (11.4%) somatotroph adenomas, 10 (7.1%) gonadotroph adenomas, 5 (3.6%) lactotroph adenomas, and 57 (40.7%) unspecified nonfunctioning PAs (unspecified NFPAs). Unspecified NFPAs refer to clinically nonfunctioning PAs from the GEO database because these tumors were not pathologically classified with pituitary transcription factor 1 or t-box transcription factor according to the 2017 World Health Organization classification (40). Therefore, the unspecified NFPAs might consist of silent pituitary adenomas (SPAs), null cell adenomas (NCAs), and clinically nonfunctioning gonadotroph adenomas. In addition, the 134 PA samples from the E-MTAB-7768 dataset were divided into 8 subtypes as follows: 27 (20.1%) corticotroph adenomas, 23 (17.2%) somatotroph adenomas, 29 (21.6%) gonadotroph adenomas, 16 (11.9%) lactotroph adenomas, 8 (6.0%) NCAs, 16 (11.9%) SPAs, 6 (4.5%) thyrotroph adenomas, and 9 (6.7%) mixed somatotroph/lactotroph adenomas. The thyrotroph adenomas and mixed adenomas in the E-MTAB-7768 dataset were excluded because no corresponding subtypes were found in the GEO dataset. Subsequently, the 140 patients with PAs from GEO datasets were set as the training cohort, and the 119 patients with PAs from the E-MTAB-7768 dataset were set as the validation cohort. The clinical parameters of the PAs, including the patient’s age and gender, tumor invasiveness, recurrence, tumor size, SSA treatment (for somatotroph adenomas), and USP8 mutation status (for corticotroph adenomas) were obtained from limited numbers of patients. The available clinical information is provided in Supplementary Table 2 (33).

### Identification of the distribution patterns of PA TIICs based on ImmuCellAI

The ImmuCellAI algorithm was used in this study to estimate the relative abundances of 24 types of immune cells from the retrieved RNA sequencing and microarray data. Compared with other algorithms, such as CIBERSORT (27), xCell (28), EPIC (29), and TIMER (30), ImmuCellAI demonstrated superior robustness and accuracy in estimating the abundance of immune cells, as verified by flow cytometry results (26). The immune cells detected by ImmuCellAI in this study included 18 subtypes of T cells, namely CD4^+^ T cells; CD8^+^ T cells; naïve CD4^+^ T cells; naïve CD8^+^ T cells; cytotoxic T (Tc) cells; exhausted T (Tex) cells; type 1 regulatory T (Tr1) cells; natural regulatory T (nTreg) cells; induced regulatory T (iTreg) cells; T-helper 1, 2, and 17 (Th1, Th2, and Th17) cells; follicular T-helper (Tfh) cells; central memory T (Tcm) cells; effector memory T (Tem) cells; natural killer T (NKT) cells; mucosal-associated invariant T (MAIT) cells; and gamma-delta T cells, as well as 6 other types of immune cells including B cells, natural killer (NK) cells, monocytes, macrophages, neutrophils, and dendritic cells (DCs).

### Identification of immunologically defined clusters of PAs through unsupervised consensus clustering

To further understand the immune cell infiltration patterns of the TME of PAs and to explore a novel immune classification of PAs based on the traditional pathological subgrouping of PAs, an unsupervised consensus clustering algorithm was applied using the ConsensusClusterPlus package (41). The clustering procedure was iterated 1000 times, and 80% of the data were sampled in each iteration. The optimal number of clusters was determined by the relative changes in the area under the cumulative distribution function (CDF) curves of the consensus score and consensus heatmap. A principal component analysis (PCA) was then performed to visualize and verify the results from the immune classification.

### Gene Set Enrichment Analysis of the immune clusters

The immune clusters were included as the population phenotypes, and a gene set enrichment analysis (GSEA) was performed to explore the related Kyoto Encyclopedia of Genes and Genomes (KEGG) and Reactome pathways and to investigate potential molecular mechanisms of immune patterns related to the tumorigenesis and progression of PAs (42). Enriched gene sets with a nominal *P* value < 0.05 and a false discovery rate q value < 0.25 were considered statistically significant.

### Correlation analysis between immune clusters and immune checkpoint molecules

The immune checkpoint molecules (ICMs) were collected from the study conducted by Charoentong et al (43) who reported 24 immunoinhibitory genes and 45 immunostimulatory genes. The expression patterns of these ICMs in the different immune clusters investigated in this study were visualized using a heatmap. To investigate whether the patterns of TIICs could be regulated by the ICMs, we performed a Pearson correlation analysis between the expression levels of ICMs and the abundances of the 24 types of immune cells. The regulatory networks between ICMs and TIICs were then visualized using Cytoscape.

### Prediction of the immunotherapy response

Tumor immune dysfunction and exclusion (TIDE) is a computational method developed in 2018 to predict the immune checkpoint blockade response based on pretreatment tumor gene profiles that integrate the expression signatures of T-cell dysfunction and T-cell exclusion to model the mechanisms of tumor immune evasion (44). We applied TIDE and an unsupervised subclass mapping method (SubMap) in this study to predict the potential immunotherapy responses of PAs (45). A Bonferroni-corrected *P* value < 0.05 was considered statistically significant.

### Statistical analysis

Normally distributed continuous variables are expressed as the means ± standard deviations, and categorical variables are expressed as numbers (percentages). The independent Student *t* test was used for the pairwise comparisons of the normally distributed variables between groups. The Mann-Whitney U test was used for the pairwise group comparisons of the nonnormally distributed variables. One-way analysis of variance and the Kruskal-Wallis test were used for the comparisons among more than 2 groups. The correlations between normally distributed variables were assessed using Pearson correlation analysis. A linear regression analysis was performed to assess the correlation between continuous variables, and the observed correlation was considered significant if both a *P* value < 0.001 and a correlation coefficient > 0.3 were obtained. All the analyses in this study were performed using R version 3.5.1, and a 2-sided *P* value < 0.05 was considered statistically significant.

## Results

### Immune landscape of the TME of PAs based on transcriptomic data

**Figure 1.**
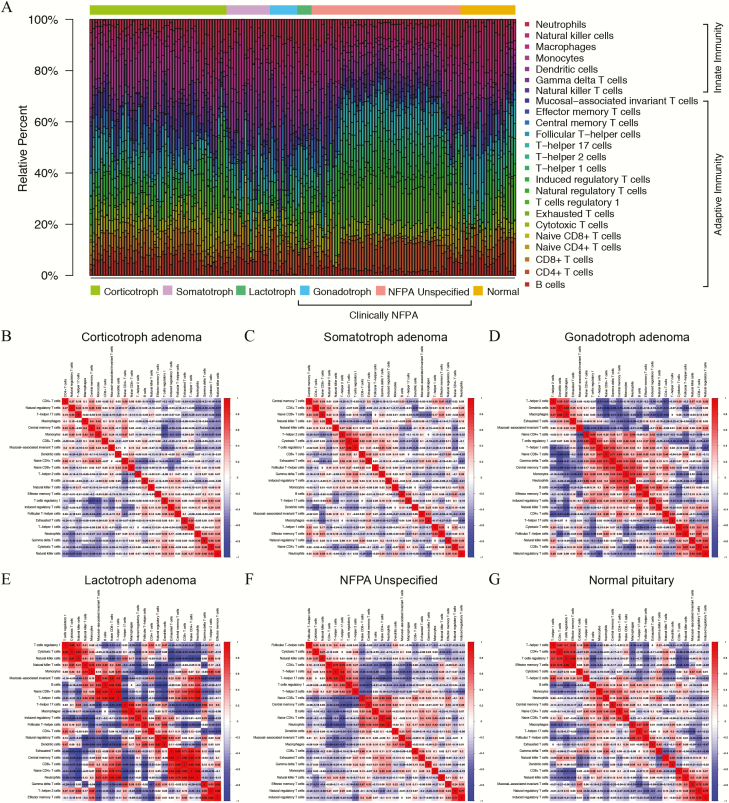
Estimation of tumor-infiltrating immune cells (TIICs) in pituitary adenomas and normal pituitaries from GEO database based on ImmuCellAI. (**A**) The relative abundances of the 24 types of TIICs are indicated by various colors. (**B-G**) Correlation analyses of the TIICs in corticotroph adenomas, somatotroph adenomas, gonadotroph adenomas, lactotroph adenomas, clinically nonfunctioning adenomas (NFPAs), and normal pituitaries. The red color represents a positive correlation, and the blue color indicates a negative correlation.

The relative abundances of 24 types of immune cells in the TME of PAs and normal pituitaries from the GEO dataset are shown in Figure 1A. Overall, the proportions of immune cells varied significantly between the normal pituitary and PA samples. Notably, the proportions of TIICs also showed marked variations among PAs of the same subtype. Correlation analyses of TIICs indicated that the CD4^+^ T-cell subsets, including CD4^+^ T cells in general as well as Tr1 cells, Th1 cells, and Tem cells, exhibited strong positive correlations with each other in normal pituitaries, whereas these correlations were attenuated in the PA samples (Fig. 1B-1G). In addition, the CD8^+^ T-cell subsets, including CD8^+^ T cells in general as well as Tc cells, MAIT cells, and Tex cells, were significantly correlated with the CD4^+^ T-cell subsets in normal pituitaries, and these correlations were attenuated in corticotroph adenomas, somatotroph adenomas, and NFPAs but amplified in gonadotroph and lactotroph PAs. In general, the correlations among TIICs in the TME of PAs compared with the normal pituitary were greatly attenuated in corticotroph and somatotroph PAs, moderately attenuated in unspecified NFPAs, and amplified in gonadotroph and lactotroph PAs.

In terms of quantitative abundances, adaptive immune cells comprised the majority of immune cells in both normal pituitaries and PAs (61.1% vs 63.2%). T cells as a whole (61.5% vs 64.8%), including the adaptive and innate T-cell subtypes, dominated both normal pituitaries and PAs.

Compared with the proportions in normal pituitaries, PAs had reduced proportions of B cells, Tex cells, Th17 cells, Tfh cells, and NK cells but increased proportions of naïve CD4^+^ T cells, naïve CD8^+^ T cells, Tr1 cells, Tcm cells, Tem cells, monocytes, and neutrophils. However, the proportions of adaptive immune cells, including CD4^+^ T cells, CD8^+^ T cells, Tc cells, nTreg cells, iTreg cells, Th1 cells, Th2 cells, and MAIT cells, as well as innate immune cells including NKT cells, gamma-delta T cells, DCs, and macrophages, in PAs did not significantly differ from those in normal pituitaries (Table 1).

**Table 1.** Comparisons of the relative abundance of 24 tumor-infiltrating immune cells among the 5 subtypes of pituitary adenomas and normal pituitaries

TIICs (%)	Total Pituitary Adenoma (n = 140)	Corticotroph (n = 52)	Somatotroph (n = 16)	Gonadotroph (n = 10)	Lactotroph (n = 5)	NFPA Unspecified (n = 57)	Normal Pituitary (n = 20)	*P*
**B cells**	4.12 ± 2.61	5.51 ± 2.55	5.78 ± 2.40	2.37 ± 2.03	4.77 ± 4.23	2.64 ± 1.47	6.15 ± 2.95	0.002
**CD4** ^**+**^ **T cells**	6.29 ± 3.83	3.82 ± 1.98	4.17 ± 3.45	9.00 ± 2.95	4.43 ± 2.93	8.83 ± 3.54	6.30 ± 3.27	0.988
**CD8** ^**+**^ **T cells**	3.15 ± 2.16	4.73 ± 2.17	2.85 ± 1.59	1.58 ± 1.15	3.21 ± 3.03	2.06 ± 1.31	3.37 ± 1.49	0.656
**Naïve CD4** ^**+**^ **T cells**	2.77 ± 3.24	3.27 ± 2.49	8.04 ± 3.72	3.31 ± 1.64	0.84 ± 0.18	0.90 ± 0.19	0.17 ± 0.04	<0.001
**Naïve CD8** ^**+**^ **T cells**	2.18 ± 2.11	2.94 ± 2.14	2.08 ± 1.30	3.31 ± 1.68	1.41 ± 0.74	1.38 ± 2.00	0.96 ± 1.26	0.001
**Cytotoxic T cells**	4.14 ± 3.27	4.80 ± 2.83	0.50 ± 0.87	1.62 ± 1.53	0.81 ± 1.22	5.30 ± 3.29	4.40 ± 3.71	0.747
**Exhausted T cells**	0.40 ± 0.94	0.54 ± 1.09	0.52 ± 1.48	0.48 ± 0.71	0.80 ± 0.82	0.18 ± 0.55	1.39 ± 1.90	0.033
**T cells regulatory 1**	7.21 ± 6.34	4.91 ± 5.57	0	3.84 ± 1.75	6.60 ± 3.13	11.9 ± 5.05	4.00 ± 6.58	0.037
**Natural regulatory T cells**	3.61 ± 2.85	4.24 ± 2.56	1.40 ± 2.10	0.53 ± 0.64	4.41 ± 5.06	4.11 ± 2.70	3.31 ± 3.55	0.673
**Induced regulatory T cells**	3.39 ± 3.22	3.32 ± 3.20	2.76 ± 2.46	2.53 ± 1.95	2.35 ± 2.81	3.86 ± 3.61	2.17 ± 2.94	0.113
**T-helper 1 cells**	4.68 ± 3.43	2.15 ± 1.97	5.77 ± 3.59	5.18 ± 2.41	4.76 ± 4.87	6.59 ± 3.09	3.31 ± 3.78	0.100
**T-helper 2 cells**	2.74 ± 2.62	2.56 ± 2.71	0.25 ± 0.36	0.33 ± 1.05	4.27 ± 4.07	3.88 ± 2.15	3.88 ± 3.82	0.210
**T-helper 17 cells**	6.36 ± 4.18	6.86 ± 4.58	6.61 ± 5.15	2.69 ± 3.96	5.03 ± 3.43	6.59 ± 3.29	8.95 ± 6.02	0.016
**Follicular T-helper cells**	3.14 ± 2.99	1.95 ± 2.25	3.96 ± 3.60	2.20 ± 3.78	5.35 ± 6.31	3.97 ± 2.47	8.38 ± 4.81	<0.001
**Central memory T cells**	2.78 ± 3.05	4.49 ± 2.94	2.36 ± 2.53	5.15 ± 2.67	0.36 ± 0.47	1.13 ± 2.30	0.17 ± 0.47	<0.001
**Effector memory T cells**	1.48 ± 1.84	0.28 ± 0.54	2.09 ± 2.43	3.32 ± 2.06	3.14 ± 2.18	1.94 ± 1.73	0.38 ± 0.82	<0.001
**Mucosal-associated invariant T cells**	4.71 ± 4.46	8.08 ± 4.00	2.57 ± 2.33	4.23 ± 3.11	3.12 ± 4.51	2.47 ± 3.57	3.83 ± 3.75	0.401
**Natural killer T cells**	3.29 ± 2.75	1.55 ± 1.66	4.38 ± 3.43	5.55 ± 2.66	2.06 ± 1.89	4.29 ± 2.54	3.98 ± 2.35	0.248
**Gamma delta T cells**	2.49 ± 2.10	2.99 ± 2.12	2.00 ± 1.85	1.08 ± 1.58	2.73 ± 2.52	2.39 ± 2.11	2.59 ± 2.08	0.841
**Dendritic cells**	7.10 ± 5.40	4.22 ± 2.56	15.77 ± 6.30	11.93 ± 7.78	4.45 ± 3.44	6.67 ± 3.34	6.39 ± 3.44	0.573
**Monocytes**	7.36 ± 5.32	12.10 ± 4.73	3.22 ± 2.16	3.04 ± 1.12	11.49 ± 2.65	4.59 ± 3.08	3.83 ± 1.89	<0.001
**Macrophages**	5.63 ± 4.81	5.11 ± 2.57	10.81 ± 7.82	10.77 ± 2.64	3.65 ± 4.56	3.92 ± 3.38	6.21 ± 4.05	0.607
**Natural killer cells**	5.84 ± 2.95	5.07 ± 2.99	4.72 ± 2.17	7.80 ± 3.48	6.43 ± 5.17	6.47 ± 2.51	13.22 ± 5.36	<0.001
**Neutrophils**	5.16 ± 3.89	4.51 ± 3.35	7.38 ± 4.09	8.16 ± 1.54	13.52 ± 4.14	3.88 ± 3.14	2.67 ± 2.02	<0.001

The *P* value indicates the variations between the total pituitary adenoma group and the normal pituitary group. “NFPA Unspecified” refers to clinically NFPAs without the 2017 World Health Organization classification, and this group might consist of silent pituitary adenomas, null cell adenomas, and gonadotroph adenomas. Abbreviations: NFPA, nonfunctioning PA; PA, pituitary adenoma; TIIC, tumor-infiltrating immune cell.

**Figure 2.**
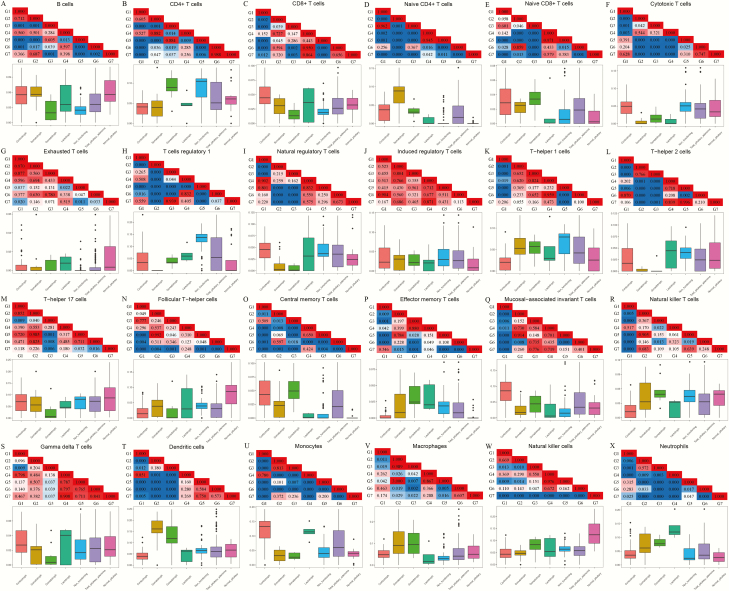
Comparisons of the abundances of 24 types of tumor-infiltrating immune cells (TIICs) among the 5 histological subtypes of pituitary adenomas (PAs), all pituitary adenomas, and normal pituitaries. Lower panel: The box plots present the fraction of each immune cell in each of the 7 groups. Upper panel: Each square of the grid represents the *P* value of the difference between 2 groups. The red color represents *P* > 0.05, whereas the blue color indicates *P* < 0.05. G1, corticotroph adenoma group; G2, somatotroph adenoma group; G3, gonadotroph adenoma group; G4, lactotroph adenoma group; G5, clinically nonfunctioning adenoma group; G6, combined PA group; G7: normal pituitary group.

Substantial heterogeneity of the distributions of different types of TIICs was found among different histological subtypes of PAs. We subsequently divided the T cells into 18 types and found that monocytes, DCs, and neutrophils were the most abundant immune cells among the 24 types of TIICs in corticotroph, somatotroph, gonadotroph, and lactotroph PAs, whereas Tr1 cells were the most common TIICs in unspecified NFPAs (Supplementary Fig. 1 (33)). The comparisons between different subtypes of PAs and normal pituitaries revealed significant differences in the proportions of different TIICs, as detailed in Figure 2. Only iTreg cells did not show a significant difference between any PA subtype and normal pituitaries. The diverse characteristics of the abundances of TIICs in each PA subtype directly reflected the differences in their immunity patterns. The distributions of 24 types of TIICs among the 6 subtypes of PAs in the validation set are shown in Supplementary Figure 2 (33).

### Clinical relevance of TIICs in PAs

**Figure 3.**
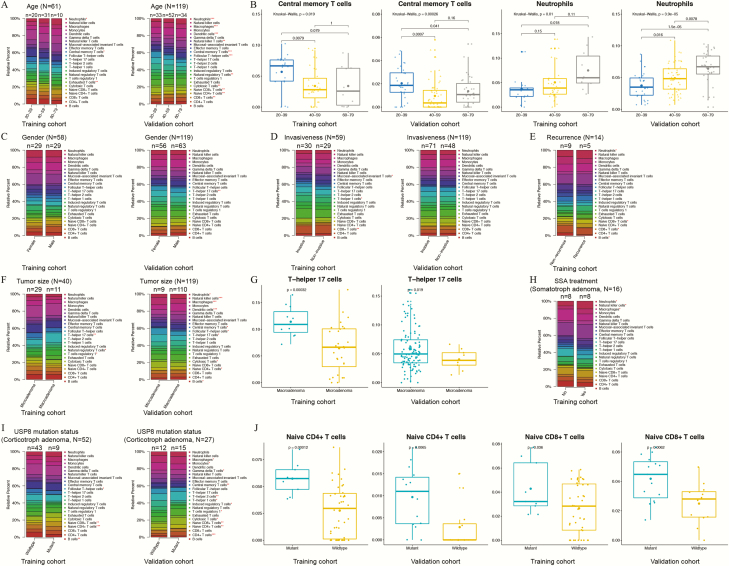
Distributions of tumor-infiltrating immune cells (TIICs) in pituitary adenomas with different clinical and pathological parameters, including patient age (**A**), patient gender (**C**), invasiveness (**D**), recurrence status (**E**), tumor size (**F**), somatostatin analog (SSA) treatment before surgery (**H**), and ubiquitin-specific protease 8 (USP8) mutation status (**I**). Left panel: Training set from multiple GEO datasets. Right panel: Validation set from the E-MATB-7768 dataset. Asterisks indicate *P* < 0.05 among groups (***, *P* < 0.001; **, *P* < 0.01; *, *P* < 0.05). (**B, G, J**) Box plots of the TIICs with significant differences between groups in both the training and validation cohorts were displayed.

The distributions of TIICs in PAs varied significantly based on different clinicopathological parameters. To strengthen the credibility of this conclusion, we investigated the correlations between clinical information and TIICs in both the training (GEO) and validation (E-MATB-7768) cohorts, and only those TIICs that showed significant differences in both datasets could be considered closely related to clinical parameters. As shown in Figures 3A and 3B, neutrophils were significantly more abundant in patients aged 60 to 79 years than in younger patients (*P* < 0.05), whereas the abundance of Tcm cells was significantly higher in patients aged 20 to 39 years than in older patients (*P* < 0.05). In the validation cohort, the gender and invasiveness of PAs did not show significant correlations with TIICs (Figs. 3C and 3D). As shown in Figures 3F and 3G, the abundance of Th17 cells was significantly higher in macroadenomas than in microadenomas (*P* < 0.05). Regarding corticotroph PAs, patients with the mutant USP8 protein tended to have higher abundances of naïve CD4^+^ T cells (*P* < 0.01) and naïve CD8^+^ T cells (*P* < 0.05) than patients with the wildtype USP8 (Figs. 3I and 3G) in both cohorts. In the GEO database, patients with recurrent PAs had lower abundances of B cells (*P* = 0.001) and CD8^+^ T cells (*P* = 0.025) and higher abundances of naïve CD8^+^ T cells (*P* = 0.025) and neutrophils (*P* = 0.012) than patients with nonrecurrent tumors, and the analysis of somatotroph PAs showed that patients who had received SSA treatment before surgery presented increased ratios of NK cells (*P* = 0.024) and neutrophils (*P* = 0.044) but reduced ratios of macrophages (*P* = 0.027) in the tumors. However, the correlations of recurrence and SSA treatment with TIICs were not verified with the validation cohort due to lack of data (Figs. 3E and 3H).

### A novel immune classification of PAs based on TIIC distributions

**Figure 4.**
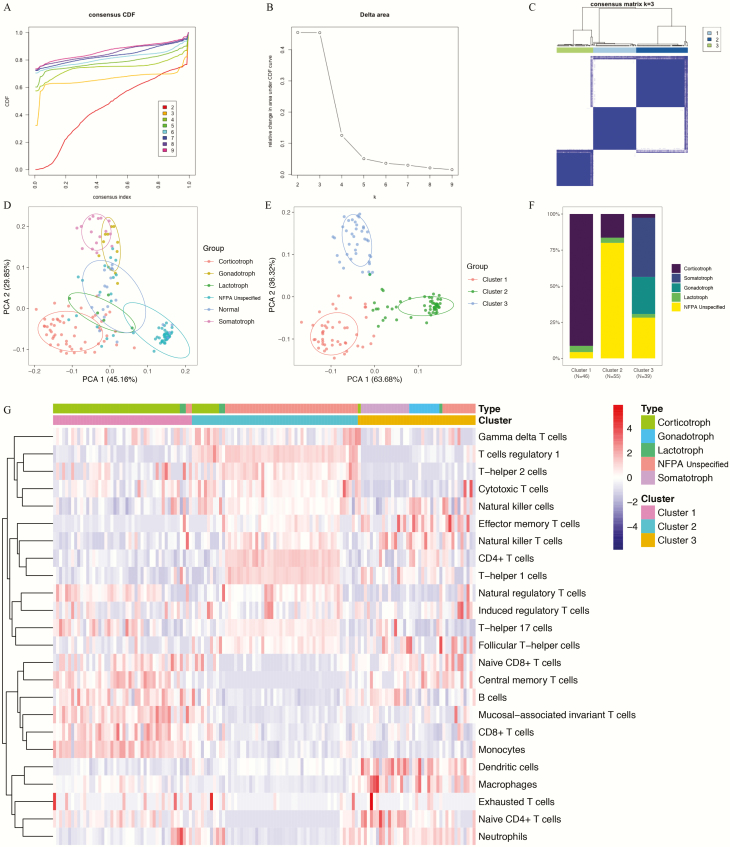
Novel immune classifications of pituitary adenomas (PAs) in the training set based on the patterns of immune cell infiltration analyzed using the unsupervised consensus clustering algorithm. (**A**) Cumulative distribution function (CDF) curves of the consensus score (k = 2-9). (**B**) Relative change in the area under the CDF curve (k = 2-9). (**C**) Consensus clustering matrix for k = 3, which was the optimal cluster number. (**D**) Principal component analysis (PCA) of the tumor-infiltrating immune cell (TIIC) patterns according to intrinsic subtyping. (**E**) PCA according to the novel immune clusters. (**F**) Distributions of the 5 intrinsic PA subtypes within the novel immune clusters. (**G**) Heatmap representing the distributions of 24 types of TIICs among the 3 novel immune clusters.

To clarify TIIC patterns based on the histological subgrouping of PAs, potential immune clusters were identified by unsupervised consensus clustering. According to the relative change in the area under the CDF curve and the consensus heatmap, the optimal number of clusters was determined to be 3 (k value = 3). All the patients from GEO cohort were divided into 3 immune clusters, and no appreciable increase in the area under the CDF curve was detected (Figs. 4A-4C). A PCA of the TIIC patterns was performed according to histological subgrouping, and this analysis demonstrated clear separations among the 5 subtypes of PAs and normal pituitaries (Fig. 4D).

A novel classification including 3 completely separated immune clusters was identified in the 140 PAs, and this classification was independent of the histological subtypes (Fig. 4E). All the PA samples were thus divided into these clusters (Fig. 4F):

- Cluster 1: 46 tumors (32.9%), including 42 (91.3%) corticotroph PAs, 2 (4.3%) lactotroph PAs, and 2 (4.3%) unspecified NFPAs
- Cluster 2: 55 tumors (39.3%) including 9 (16.4%) corticotroph PAs, 2 (3.6%) lactotroph PAs, and 44 (80%) unspecified NFPAs
- Cluster 3: 39 tumors (27.8%) including 1 (2.6%) corticotroph PA, 1 (2.6%) lactotroph PA, 11 (28.2%) unspecified NFPAs, all 16 (41.0%) somatotroph PAs, and all 10 (25.6%) gonadotroph PAs.

Each immune cluster presented unique patterns of TIIC distributions (Fig. 4G). The detailed TIIC distributions among the clusters are summarized in Table 2 and visualized in Supplementary Figure 3 (33).

**Table 2.** Comparisons of the Relative Abundance of 24 Tumor-Infiltrating Immune Cells (TIICs) Among the Three Immune Clusters of Pituitary Adenomas

TIICs (%)	Cluster 1 (n = 46)	Cluster 2 (n = 55)	Cluster 3 (n = 39)	*P* (total)	*P* (1, 2)	*P* (1, 3)	*P* (2, 3)
**B cells**	5.87 ± 2.41	2.66 ± 1.84	4.14 ± 2.56	<0.001	<0.001	0.006	0.009
**CD4** ^**+**^ **T cells**	3.90 ± 1.97	8.78 ± 3.56	5.60 ± 3.84	<0.001	<0.001	0.017	<0.001
**CD8** ^**+**^ **T cells**	4.96 ± 2.15	2.28 ± 1.33	2.23 ± 1.76	<0.001	<0.001	<0.001	0.998
**Naïve CD4** ^**+**^ **T cells**	3.33 ± 2.55	0.81 ± 1.71	4.86 ± 4.01	<0.001	<0.001	0.013	<0.001
**Naïve CD8** ^**+**^ **T cells**	2.99 ± 2.07	1.05 ± 1.61	2.80 ± 2.11	<0.001	<0.001	0.651	<0.001
**Cytotoxic T cells**	4.09 ± 2.66	5.69 ± 2.59	2.02 ± 3.60	<0.001	0.007	0.001	<0.001
**Exhausted T cells**	0.52 ± 0.99	0.32 ± 0.83	0.36 ± 0.10	0.516	0.267	0.418	0.827
**T cells regulatory 1**	2.77 ± 2.58	14.22 ± 2.48	2.56 ± 2.29	<0.001	<0.001	0.740	<0.001
**Natural regulatory T cells**	4.64 ± 2.64	3.99 ± 2.49	1.84 ± 1.81	<0.001	0.214	<0.001	<0.001
**Induced regulatory T cells**	2.93 ± 3.06	3.54 ± 3.13	3.73 ± 3.54	0.479	0.345	0.258	0.784
**T-helper 1 cells**	2.54 ± 2.47	6.41 ± 3.30	4.77 ± 3.24	<0.001	<0.001	0.001	0.011
**T-helper 2 cells**	2.71 ± 2.60	4.30 ± 1.87	0.56 ± 0.10	<0.001	0.008	<0.001	<0.001
**T-helper 17 cells**	7.02 ± 4.82	7.34 ± 2.51	4.19 ± 4.04	<0.001	0.688	0.001	<0.001
**Follicular T-helper cells**	1.69 ± 1.40	4.00 ± 2.05	3.74 ± 3.70	<0.001	<0.001	0.001	0.660
**Central memory T cells**	4.96 ± 2.78	0.32 ± 0.86	3.67 ± 2.92	<0.001	<0.001	0.010	<0.001
**Effector memory T cells**	0.41 ± 0.96	1.53 ± 1.18	2.68 ± 2.53	<0.001	0.001	<0.001	0.001
**Mucosal-associated invariant T cells**	8.85 ± 4.04	2.16 ± 2.38	3.45 ± 3.69	<0.001	<0.001	<0.001	0.069
**Natural killer T cells**	1.89 ± 2.22	3.69 ± 2.28	4.38 ± 3.25	<0.001	0.001	<0.001	0.204
**Gamma delta T cells**	2.48 ± 1.87	2.89 ± 2.15	1.93 ± 2.11	0.091	0.325	0.226	0.079
**Dendritic cells**	3.62 ± 2.10	5.79 ± 2.35	13.03 ± 6.37	<0.001	0.006	<0.001	<0.001
**Monocytes**	13.52 ± 3.57	4.81 ± 2.88	3.68 ± 2.71	<0.001	<0.001	<0.001	0.084
**Macrophages**	5.23 ± 2.61	2.86 ± 1.53	10.01 ± 6.51	<0.001	0.002	<0.001	<0.001
**Natural killer cells**	4.50 ± 2.90	6.74 ± 2.03	6.16 ± 3.54	<0.001	<0.001	0.007	0.329
**Neutrophils**	4.64 ± 3.98	3.85 ± 3.36	7.62 ± 3.37	<0.001	0.269	<0.001	<0.001

*P* value (total) refers to the variation among the 3 clusters. *P* (1, 2) indicates the variation between clusters 1 and 2. *P* (1, 3) shows the variation between clusters 1 and 3. *P* (2, 3) indicates the variation between clusters 2 and 3. Abbreviation: TIIC, tumor-infiltrating immune cell.

Cluster 1 presented significantly higher abundances of B cells, CD8^+^ T cells, Tcm cells, MAIT cells, and monocytes and significantly lower abundances of CD4^+^ T cells, Th1 cells, Tfh cells, Tem cells, NKT cells, DCs, and NK cells than the other 2 clusters. Cluster 2 presented significantly higher abundances of CD4^+^ T cells, Tc cells, Tr1 cells, Th1 cells, and Th2 cells and significantly lower abundances of B cells, naïve CD4^+^ T cells, naïve CD8^+^ T cells, Tcm cells, and macrophages than the other 2 clusters. In addition, cluster 3 had significantly higher abundances of naïve CD4^+^ T cells, Tem cells, DCs, macrophages, and neutrophils and significantly lower abundances of Tc cells, nTreg cells, Th2 cells, and Th17 cells than the other 2 clusters. Overall, the TIICs that showed the most variable abundances among the immune clusters were B cells, CD4^+^ T-cell subsets, Tc cells, effector and memory T cells (attributed to both CD4^+^ and CD8^+^ T-cell subsets), DCs, and macrophages.

### GSEA of the 3 immune clusters

A GSEA was performed to explore the pathways and molecular mechanisms related to the novel immune classification, and the results revealed that all 3 immune clusters were enriched in KEGG and Reactome pathways related to tumor development, progression, metabolism, and immune state. As shown in Supplementary Table 3 (33), cluster 1 was enriched in 28 pathways (eg, signaling by the B-cell receptor, Toll-like receptor signaling pathway, B- and T-cell receptor signaling pathways, and innate immune system), cluster 2 was enriched in 33 pathways (eg, cell cycle, mitotic prophase, apoptosis, NK cell-mediated cytotoxicity, T-cell receptor signaling pathway, and pathways in cancer), and cluster 3 was enriched in 19 pathways (eg, adaptive immune system, calcium signaling pathway, B-cell receptor signaling pathway, focal adhesion, and NK cell-mediated cytotoxicity).

### The novel immune clusters of PAs predict potential immunotherapy responses

**Figure 5.**
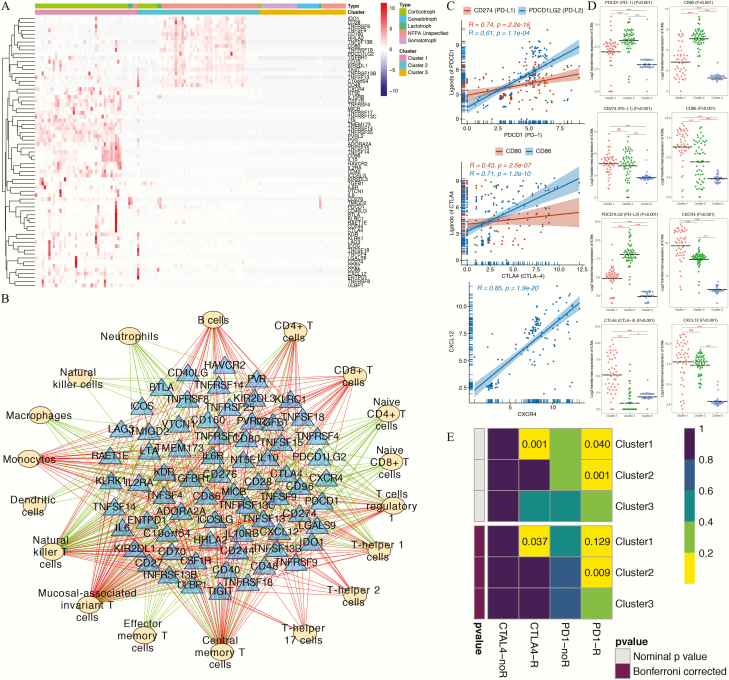
Correlation analyses between immune patterns and immune checkpoint molecules (ICMs). (**A**) Heatmap representing the expression profiles of 69 immune checkpoint genes among the 3 novel immune clusters and 5 histological pituitary adenoma (PA) subtypes. (**B**) Regulatory networks of ICMs and tumor-infiltrating immune cells (TIICs). The yellow ovals represent the TIICs, and the blue triangles represent the ICMs. The green/red lines indicate negative/positive correlations between the ICMs and the TIICs. (**C**) Regression analyses between immune checkpoint receptor-ligand pairs. (**D**) Expression of immune checkpoint receptors and ligands among the 3 immune clusters. (**E**) The subclass mapping analysis demonstrated that immune cluster 1 was more sensitive to anti-CTLA4 therapy (Bonferroni-corrected *P* = 0.037) and that immune cluster 2 was more sensitive to anti–programmed cell death protein 1 (PD1) therapy (Bonferroni-corrected *P* = 0.009). In this figure, “R” is short for immunotherapy respondent.

The expression profiles of 69 immune checkpoint genes reported by Charoentong et al (43) were collected and are displayed in Figure 5A. The results showed that most of the ICMs were upregulated in the PAs belonging to cluster 1, whereas the remaining ICMs, mainly immunostimulatory genes, were upregulated in the PAs belonging to cluster 2. Surprisingly, almost all of the ICMs were notably downregulated in the PAs classified into cluster 3.

Strong correlations were found between PD1 (PDCD1) and PD-L1 (CD274)/PD-L2 (PDCD1LG2), between CTLA4 and CD80/CD86, and between CXCR4 and CXCL12 (Fig. 5C) in PAs. Compared with clusters 2 and 3, cluster 1 presented significantly increased expression levels of the immune checkpoint receptor CTLA4 and its corresponding ligand CD86. In contrast, cluster 2 presented significantly increased expression levels of PD1 and PD-L2 than did clusters 1 and 3 (Fig. 5D).

The likelihood of an immunotherapy response was then predicted for each immune cluster using the TIDE algorithm and subclass mapping analysis. The TIDE results predicted that clusters 1 (41.3%, 19/46) and 2 (40.0%, 22/55) were more likely to respond to immunotherapy than cluster 3 (25.6%, 10/39). The subclass mapping results indicated that cluster 1 was more sensitive to CTLA4 inhibitors (Bonferroni-corrected *P* = 0.037) and that immune cluster 2 was more sensitive to PD1 inhibitors (Bonferroni-corrected *P* = 0.009) (Fig. 5E). These findings were consistent with the expression levels of immune molecules in the 3 clusters and confirmed the clinical significance of this novel immune classification for predicting immunotherapy responses.

A subsequent correlation analysis between the expression levels of ICMs and the abundance levels of the 24 types of immune cell revealed that 18 TIICs exhibited significant correlations with 69 checkpoint genes (Supplementary Table 4 (33)). A total of 456 ICM-TIIC interaction pairs, including 263 positive correlations and 193 negative correlations, were visually represented in the regulatory network (Fig. 5B). Notably, the results showed that naïve CD4^+^ T cells, naïve CD8^+^ T cells, Tem cells, DCs, macrophages, NK cells, and neutrophils were negatively regulated by ICMs and that CD8^+^ T cells, Th2 cells, and Th17 cells were positively regulated by ICMs.

### External validation of the novel immune clusters of PAs

**Figure 6.**
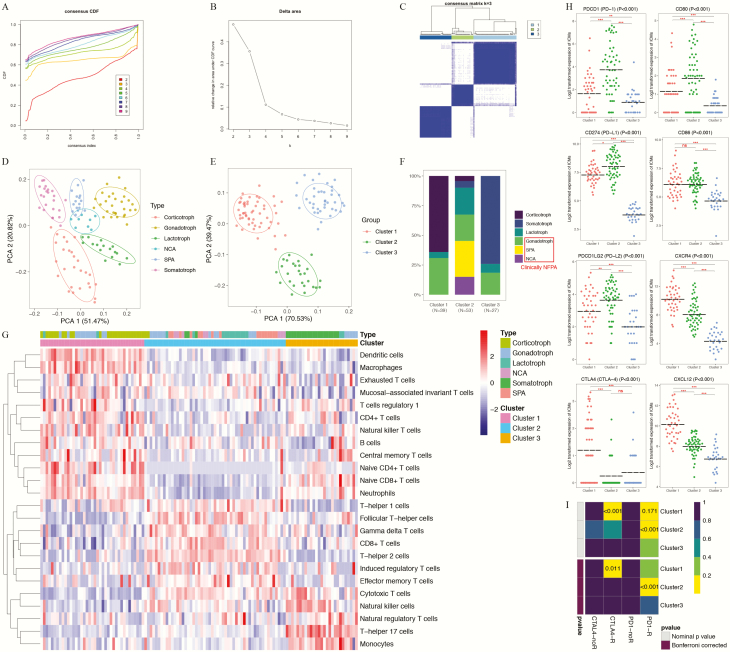
Novel immune classifications of pituitary adenomas (Pas) in the validation set based on the patterns of immune cell infiltration analyzed using the unsupervised consensus clustering algorithm. (**A**) Cumulative distribution function (CDF) curves of the consensus score (k = 2-9). (**B**) Relative change in the area under the CDF curve (k = 2-9). (**C**) Consensus clustering matrix for k = 3, which was the optimal cluster number. (**D**) Principal component analysis (PCA) of the tumor-infiltrating immune cells (TIIC) patterns according to histological subtyping. (**E**) PCA according to the novel immune clusters. (**F**) Distributions of the 6 PA subtypes within the novel immune clusters. (**G**) Heatmap indicating the distributions of 24 types of TIICs among the 3 immune clusters. (**H**) Expression patterns of immune checkpoint receptors and ligands among the 3 immune clusters. (**I**) The subclass mapping analysis demonstrated that immune cluster 1 was more sensitive to anti-CTLA4 therapy (Bonferroni-corrected *P* = 0.011) and that immune cluster 2 was more sensitive to anti–programmed cell death protein 1 (PD1) therapy (Bonferroni-corrected *P* < 0.001). In this figure, “R” is short for immunotherapy respondent.

To verify the reliability of the novel immune classification of PAs in different patient populations, we applied the E-MATB-7768 dataset (36) as the validation cohort. As shown in Figures 6A-6C, the 119 PAs in the validation set can also be classified into 3 clusters using similar methods. The PCA results also suggested clear separations among the 6 subtypes of PAs (Fig. 6D) and clear separations among 3 immune clusters (Fig. 6E). Based on the classification of gonadotroph PAs, SPAs, and NCAs as clinically NFPAs, the proportion of each histological subtype within each immune cluster in the validation set was basically equivalent to that in the training set (Fig. 6F).

- Cluster 1: 39 tumors (32.8%), including 25 (64.1%) corticotroph PAs, 2 (5.1%) lactotroph PAs, and 12 (30.8%) gonadotroph PAs
- Cluster 2: 53 tumors (44.5%), including 2 (3.8%) corticotroph PAs, 3 (5.7%) somatotroph PAs, 12 (22.6%) lactotroph PAs, 12 (22.6%) gonadotroph PAs, 16 (30.2%) SPAs, and 8 (15.1%) NCAs
- Cluster 3: 27 tumors (22.7%), including 20 (74.1%) somatotroph PAs, 2 (7.4%) lactotroph PAs, and 5 (18.5%) gonadotroph PAs.

In addition, the expression patterns of TIICs in each immune cluster in the validation cohort were exactly consistent with the findings of the GEO cohort (Fig. 6G). The TIICs that exhibited the most variable abundances among the 3 immune clusters in the validation cohort were B cells, CD4^+^ T-cell subsets, Tc cells, effector and memory T cells (attributed to both CD4^+^ and CD8^+^ T-cell subsets), DCs, and macrophages (Supplementary Fig. 4 (33)), which were generally consistent with the results obtained with the training cohort.

Based on the ICMs, cluster 1 also exhibited significant upregulation of CTLA4, CXCR4, and CXCL12, and cluster 2 showed significant upregulation of PD1, PD-L1, PD-L2, and CD80 (Fig. 6H). In contrast, cluster 3 mostly showed significantly lower expression of these ICMs than clusters 1 and 2. The likelihood of showing a response to immunotherapies was then predicted for each immune cluster in the validation cohort using the TIDE algorithm and subclass mapping analysis. In the validation set, clusters 1 (43.6%, 17/39) and 2 (50.9%, 27/53) tended to exhibit a higher response rate than did cluster 3 (33.3%, 9/27), which was consistent with the results obtained with the training set. The subclass mapping analysis suggested that cluster 1 was more sensitive to anti-CTLA4 therapies (Bonferroni-corrected *P* = 0.011) and that cluster 2 was more sensitive to anti-PD1 therapies (Bonferroni-corrected *P* < 0.001) (Fig. 6I). All these findings strongly suggested that the novel immune classification of PAs was robust and reliable and validated the clinical significance of this classification for predicting immunotherapy responses.

## Discussion

In this study, we characterized the distributions of 24 types of TIICs in the TME of PAs by analyzing transcriptomic data using the ImmuCellAI algorithm and explored the clinical relevance of the TIICs. We also identified 3 immune clusters based on the TIIC abundances across PAs independent of the intrinsic histological subgroups. Each immune cluster obtained using this novel classification had unique features related to immune checkpoint expression and exhibited multiple correlations with pathways related to tumor development, progression, and immunotherapy responses. To the best of our knowledge, this study provides the first immune classification related to immunotherapy responses, and this classification lays a foundation for further immune studies on PAs and sheds new light on the strategy of immunotherapy for PAs.

The tumor behavior is modulated by the infiltrating immune cells in the TME. Innate and adaptive TIICs suppress tumor growth by recognizing antigens expressed on the surfaces of tumor cells, whereas tumor cells show resistance to antitumor immune responses by inactivating immune cells and dysregulating immune checkpoint pathways, which are mostly related to T cells (17, 25). Dysfunctional T cells can be inactivated effectively by immunotherapy (46). Anti-PD1 and anti-CTLA4 therapies have been used to boost antitumor immune responses and have provided significant improvements in outcomes for various cancers, particularly advanced melanomas (15, 47).

The first reported case of PA treated with checkpoint inhibitors was a woman with a corticotroph PA and liver metastasis (48). Surgeries, radiotherapy, and medical treatments all failed in this patient prior to immunotherapy. Fortunately, after 5 cycles of treatments with both the anti-PD1 antibody nivolumab and the anti-CTLA4 antibody ipilimumab, the volumes of the PA and the main liver metastasis and the level of adrenocorticotropic hormone showed marked decreases. In contrast, the recently published second case of immunotherapy was unsuccessful, as demonstrated by a lack of improvement after the use of the anti-PD1 antibody pembrolizumab (49). For this second patient, although the authors demonstrated that mismatch repair deficiency might be 1 possible reason for the unsatisfactory efficacy, we hypothesize that these 2 cases might have had different distribution patterns of TIICs and ICMs that contributed to their opposing responses to immunotherapy. The different therapies, that is, combined anti-CTL4 and anti-PD1 therapies in the responsive case and only anti-PD1 in the resistant case, might be the other reason for different immunotherapy outcomes.

In our analysis, the distributions of 24 types of TIICs exhibited high heterogeneity across different histological subgroups of PAs. Even within subgroups, the TIIC distributions showed marked differences among cases. This phenomenon of heterogeneous immune infiltration in the TME has also been detected in several other tumors (50-53).

The major immune cell populations in glioblastomas and meningiomas are innate immune cells, mostly macrophages (54, 55). However, authors of studies on the TIIC distributions in PAs have not reached an agreement. Marques et al (56) reported that macrophages identified in an immunohistochemical analysis using the marker CD68 were more abundant in PAs than in normal pituitaries. Yeung et al (20) analyzed the gene expression data of PAs and demonstrated that CD4^+^ memory T cells and M2 macrophages were the most common types of TIICs in PAs and that CD8^+^ T cells dominated the corticotroph PA microenvironment. As shown in this study, most immune infiltrates were adaptive immune cells, which accounted for more than 60% of all the TIICs in PAs. The analysis of different histological subtypes of PAs revealed that nonfunctioning PAs had a higher abundance of adaptive immune cells and a lower abundance of innate immune cells than secretory PAs. Compared with innate immune cells, T cells as a whole dominated the TMEs of all PA subtypes.

Determining the differences in the TIIC composition of the TMEs of tumors with different clinical features is critical for obtaining a comprehensive understanding of tumor biology (57). However, the clinical relevance of the TIIC distributions in PAs remains under exploration. Lu et al (14) assessed the immune infiltrates in 35 PAs and demonstrated that the macrophage abundance was positively correlated with the tumor size and aggressiveness. In the present study, however, no correlations were detected between the distribution of macrophages and the size or aggressive behavior of tumors. Instead, we found that the tumor size and patient age presented different correlations with the distribution of TIICs, mostly neutrophils and T cells. Some of these results are novel to those of previous studies (13, 18, 19, 58).

The USP8 mutation is unique to corticotroph adenomas among all subtypes of PAs, and cells with the USP8 mutation secrete higher levels of adrenocorticotropic hormone than cells with the wildtype USP8 protein (59). In addition, a study involving RNA sequencing and enrichment analysis revealed that USP8-mutated PAs exhibit a low epithelial-to-mesenchymal transition rate and are clinically less invasive than USP8 mutation-negative PAs (37). Moreover, the protease USP8 was found to be crucial for T-cell development and homeostasis in a mouse model (60). In the present analysis, the USP8 mutation was found to be correlated with the intratumoral abundance of TIICs, particularly adaptive immune T cells. From the result using both databases, corticotroph PAs with mutated USP8 contained more naïve CD4^+^ and CD8^+^ T cells than PAs without mutated USP8, which might suggest a potential mechanism linking immune cell infiltration, tumor invasiveness, and hormone secretion, but this relationship requires further confirmation. These findings are interesting and might provide a clue for the different treatment outcomes of PAs with different USP8 mutation statuses.

The KEGG and Reactome databases have been widely used to provide intuitive bioinformatics tools that can provide pathway-related knowledge that can support genetic analyses. GSEA, which allows the interpretation of genomic results to provide insights into biological mechanisms, was performed with these 2 databases in this study. The results showed that each cluster was enriched with diverse pathways, including pathways related to the immune state, immune interactions, the cell cycle, tumor proliferation, and apoptosis, which indicated that these immune clusters exhibit different potential biological correlations.

Thorsson et al (17) identified 6 immune subtypes across 33 types of cancers and proposed that this novel classification might guide treatment approaches independent of the histological type. In this study, we identified 3 novel immune clusters across PAs according to the TIIC abundance levels independent of the traditional histological PA classification. Each immune cluster had unique features related to immune checkpoint expression and presented distinctive correlations with diverse pathways related to tumor behavior. These findings indicate that these novel clusters possess different immune capacities and features, and these differences might contribute to the varied responses of the clusters to specific immunotherapies and traditional treatments, as particularly demonstrated by the responses of PAs belonging to the same histological subtype but different immune clusters.

Most of the ICMs were notably upregulated in the PAs belonging to cluster 1; thus, we called cluster 1 the “hot immune cluster” (Fig. 5A) and predicted that the PAs belonging to this cluster would have certain checkpoints potentially available for checkpoint-targeted immunotherapy. In contrast, we named cluster 3 the “cold immune cluster” because ICMs tended to be downregulated in this cluster, and we speculated that the PAs belonging to cluster 3 might exhibit low responsiveness to immunotherapy due to a lack of targetable immune checkpoints. After being confirmed using the TIDE algorithm and unsupervised subclass mapping methods, our findings clearly demonstrated the potential of our novel immune classification to provide new evidence for tumor-targeted treatments.

We investigated the expression of key receptor-ligand pairs in these 3 immune clusters. PD1 has 2 ligands, namely PD-L1 and PD-L2 (61), and CTLA-4 also has 2 ligands, namely CD80 and CD86 (62). Because PD1 and PD-L2 expression is higher in cluster 2 than in the other 2 clusters, we hypothesized that the antitumor immune responses in PAs belonging to cluster 2 might be largely suppressed by the PD1/PD-L2 pathway and that this group of patients might be particularly good candidates for anti-PD1 therapy. Moreover, due to the high expression of CTLA4 and CD86 in cluster 1, we hypothesized that the antitumor immune responses in PAs belonging to cluster 1 might be largely suppressed by the CTLA4/CD86 pathway. In addition to being the ligand for CTLA4, CD86 also acts as an immune-activation ligand for CD28 on other cytotoxic immune cells (63, 64) and is therefore not an optimal alternative for immunotherapy. Thus, patients classified in cluster 2 might be good candidates for anti-CTLA4 therapy. Because corticotroph PAs can be divided into clusters 1, 2 and 3, we speculate that the different responses of the 2 published cases of refractory corticotroph PAs to immunotherapy (48, 49) might be attributable to different immune clusters with disparate patterns of immune checkpoint expression and TIIC distribution. Additionally, because PD1/PD-L2 and CTLA4/CD86 are expressed on all immune clusters, combination treatment with both anti-PD1 and anti-CTLA4 might be more effective than the single treatments for corticotroph PAs. Moreover, we hypothesize that somatotroph PAs, most of which were classified in the cold immune cluster (cluster 3), might exhibit a less satisfactory immunotherapy response than other PAs.

In conclusion, by analyzing the transcriptomic profiles of PAs using the ImmuCellAI algorithm, we characterized the immune profile of PAs, investigated its clinical relevance, and established a novel immune classification with 3 independent clusters. These immune clusters were distinct in terms of the distributions of immune infiltrates in the TME of PAs, the expression levels of ICMs that are used for immunotherapeutic targets, and the expression of the pathways related to the development, progression, and immunotherapy responses of tumors. These findings regarding the TME of PAs might provide new insights that can be used to guide strategies for immunotherapy for PAs. Further studies are needed to confirm these conclusions.

## Abbreviations

CDF: cumulative distribution function
CTLA4: cytotoxic T-lymphocyte-associated antigen
DC: dendritic cell
GSEA: gene set enrichment analysis
ICM: immune checkpoint molecule
ImmuCellAI: immune cell abundance identifier
iTreg: induced regulatory T
KEGG: Kyoto Encyclopedia of Genes and Genomes
MAIT: mucosal-associated invariant T
NCA: null cell adenoma
NFPA: nonfunctioning PA
NKT: natural killer T
nTreg: natural regulatory T
PA: pituitary adenoma
PCA: principal component analysis
PD1: programmed cell death protein 1
PD-L2: programmed death-ligand 1
RNA: ribonucleic acid
SPA: silent pituitary adenoma
SSA: somatostatin analog
Tc: cytotoxic T
Tcm: central memory T
Tem: effector memory T
Tex: exhausted T
Tfh: follicular T helper
Th: T helper
TIDE: tumor immune dysfunction and exclusion
TIIC: tumor-infiltrating immune cell
TME: tumor microenvironment
Tr1: type 1 regulatory T
USP8: ubiquitin-specific protease 8

## Additional Information

***Data Availability:*** The transcriptomic data of the PA and normal pituitary samples are available to be downloaded from the GEO database (GSE136781, GSE132982, GSE119063, GSE93825, GSE51618, GSE46311, and GSE26966) and the ArrayExpress database (E-MTAB-7768).

***Disclosure Statement:*** The authors have nothing to disclose.

The supplementary files are available at https://doi.org/10.6084/m9.figshare0.12545954.v1.

***Statement of Ethics:*** Approval of our study from the ethics committee was not necessary because the data were obtained from publicly available databases.
